# Adaptation of the brainwriting premortem technique to inform the co-creation of COVID-19 testing strategies in underserved communities in South San Diego

**DOI:** 10.1186/s12913-023-10341-w

**Published:** 2024-01-03

**Authors:** Borsika A. Rabin, Kelli L. Cain, Lawrence O. Ayers, Angel Lomeli, Arleth Escoto, Maria Linda Burola, Melanie Aguilar, Stephenie Tinoco Calvillo, Breanna Reyes, Linda Salgin, Robert Tukey, Louise C. Laurent, Nicole A. Stadnick

**Affiliations:** 1 Herbert Wertheim School of Public Health and Human Longevity Science, University of California San Diego, La Jolla, CA USA; 2 UC San Diego Altman Clinical and Translational Research Institute Dissemination and Implementation Science Center, University of California San Diego, La Jolla, CA USA; 3 Department of Obstetrics, Gynecology, and Reproductive Sciences, University of California San Diego, La Jolla, CA USA; 4 San Ysidro Health, San Diego, CA USA; 5 Superfund Research Center, University of California San Diego, La Jolla, CA USA; 6 Department of Pharmacology, University of California San Diego, La Jolla, CA USA; 7 Department of Psychiatry, University of California San Diego, La Jolla, CA USA; 8 Child and Adolescent Services Research Center, San Diego, CA USA

**Keywords:** Brainwriting premortem, Implementation science, Co-creation, COVID-19, Underserved communities, Partner engagement, Qualitative methods, Rapid adaptations

## Abstract

**Introduction:**

Meaningful engagement of partners in co-creating and refining health-related programs can increase the initial uptake, sustained implementation, broad reach, and effectiveness of these programs. This is especially important for underserved communities where resources are limited and need to be prioritized. Brainwriting premortem is a novel qualitative approach to partner engagement that combines the strengths of individual idea generation with the concept of premortem exercise that addresses failure points prior to the implementation of new programs.

**Methods:**

An adapted form of brainwriting premortem was used to inform iterative refinements to a COVID-19 testing program at a Federally Qualified Health Center (FQHC) in San Diego. Patients and providers from the FQHC participated in interviews at two time points (early- and mid-implementation of the program). Interview data were transcribed, translated, and analyzed using a rapid qualitative approach. Key themes and sub-themes were identified and used to inform refinements to the program.

**Results:**

A total of 11 patients (7 Spanish- and 4 English-speaking) and 8 providers participated in the brainwriting premortem interviews. Key themes related to possible reasons for COVID-19 testing program failure: advertising/sharing information; access to testing; handling of test results; staff and patient safety; patient beliefs and views regarding the SARS-CoV-2 virus; and COVID-19 testing options offered. Proposed solutions were offered for the key failures except for patient beliefs and views regarding the SARS-CoV-2 virus. Additional solutions offered were related to education, physical operations, and recruitment strategies. Real-time changes to the program flow and components were made in response to 7 suggestions from patients and 11 from providers. Changes related to the process of returning results were the most common, and included sending results via email with distinct workflows based on the test result.

**Conclusion:**

The implementation of the adapted brainwriting premortem technique allowed us to incorporate the perspective of key partners in the delivery and iterative refinement of the COVID-19 testing program. This was an effective tool in the context of an FQHC and can be a promising and approach to incorporate iterative input from patients and providers to ensure successful program implementation. Future studies, particularly those requiring rapid response to public health emergencies, should consider the use of this technique.

**Supplementary Information:**

The online version contains supplementary material available at 10.1186/s12913-023-10341-w.

## Contributions to the literature

- Meaningful engagement of partners in co-creating and refining programs to address important public health priorities can greatly increase the initial uptake, sustained implementation, broad reach, and effectiveness of these programs.
- Our paper describes an adapted form of an innovative approach, brainwriting premortem to engage patients and providers in a federally qualified health center to refine the workflow for a COVID-19 testing program.
- The adapted brainwriting premortem proved to be a feasible approach to implement at multiple time-points to gain perspectives from multiple partners and supported an iterative improvement of the program protocol.

## Introduction

Widening health disparities among underservedcommunities such as Latino/a, Black, Indigenous, and people of color (BIPOC) were experienced with the COVID-19 pandemic [1]. These communities showed lower testing and vaccination rates compared to white individuals in the United States and were significantly more likely to experience mortality and morbidity from COVID-19 [2]. Further inflating the likelihood of ongoing transmission in these communities was reduced access to testing resources [3–8]. Meaningful engagement of partners in co-creating and refining programs to address important public health priorities can greatly increase the initial uptake, sustained implementation, broad reach, and effectiveness of these programs [9, 10]. Engagement is especially important when programs are offered in settings that serve historically under-represented and culturally diverse communities, often with limited resources that need to be prioritized [11, 12]. When responding to public health emergencies, the need for rapid action in the context of uncertainty and lack of definitive evidence means that program development is best undertaken through an iterative approach. The initial program is created based on the best available evidence and program elements are refined over time as additional knowledge is gained and feedback is received on implementation strategies from partners [13–15].

The Community-driven Optimization of COVID-19 testing to Reach and Engage underserved Areas for Testing Equity—in Women and Children (CO-CREATE) program is a partnership between investigators in an academic institution and a Federally Qualified Health Center (FQHC). The CO-CREATE program was designed to be responsive to the needs of the community and offers no-cost COVID-19 testing to patients and community members at the clinic site of the partnering (FQHC). Throughout our work we engaged with a multidisciplinary Community and Scientific Advisory Board to guide our program development and implementation [11, 16].

A number of approaches are available to support partner engagement [17] (e.g., intervention mapping and implementation mapping [18], human-centered design approaches [19], qualitative systems mapping [20], rapid process improvement workshops [21], etc. In many cases, the use of multiple engagement strategies is required to achieve the most comprehensive understanding of perspectives from all partners, which can then support the creation of programs with the best fit to local priorities and resources [22]. There are few innovative techniques that seek input from diverse partners at multiple time points and allow for iterative and rapid improvement of a program. These techniques support bringing programs into practice rapidly that highlight the stakeholder’s voice. Brainwriting and the premortem technique are two innovative engagement methods that are gaining attention in public health implementation [23].

Brainwriting was developed in the context of marketing to provide an alternative to the traditional group-based brainstorming approach [24]. This technique involves asynchronous brainstorming where people contribute ideas independently using a method of writing down all of their ideas on a topic in a short period of time while in a group setting. Individual ideas are then shared with others in the group to expand on or to add new ideas. Brainwriting combines the strength of individual idea generation with the strength of group exchange [24]. The premortem technique has been used in the creation of new products, technology, and programs to predict risk for failure and prevent such failures from happening prior to launch [25]. The process involves assuming that the new product, technology, or program failed and works backwards to identify factors that might have led to this failure. A novel technique that combines these two approaches, brainwriting premortem, builds on a combination of individual wisdom and group problem-solving to identify potential failure points for the implementation of a program and possible solutions for these failure points [23]. Brainwriting premortem has been used recently in the context of implementation science to inform the refinement of interventions based on input from multiple partners [26–28]. While traditional approaches to the brainwriting premortem include in-person group-based activities, this was not feasible during the early stages of the COVID-19 pandemic for members of underserved communities or frontline healthcare providers due to the increased care demands on families and on providers at clinics. Our team adapted the traditional brainwriting premortem approach to an individually engaged format, both in-person and virtually, where community members and frontline providers provided their perspectives on a COVID-19 testing program at two separate time points. This paper describes the approach, key findings from the process, and how this information was used to refine our testing program.

## Methods

CO-CREATE is funded through the NIH Rapid Acceleration of Diagnostics-for Underserved Populations (RADx-UP) initiative to understand practices, barriers, and facilitators to the access and uptake of COVID-19 testing and follow-up for members residing in an underserved community in South San Diego near the U.S./Mexico border. The primary deliverable of CO-CREATE was the design and implementation of a no-cost COVID-19 testing program that was responsive to the needs of the community. Data for this manuscript were extracted from the larger CO-CREATE research study (described in the next section).

### CO-CREATE COVID-19 testing program

The CO-CREATE program was established in May 2021 and continues to offer free COVID-19 testing to patients and community members at the clinic site of the partnering FQHC. Community members can decide to participate in the research component of the program or simply access the testing services. Participating in the CO-CREATE research component involves completing a survey and a COVID-19 test. Upon arrival to the testing site, interested participants register to be part of the study by providing a valid form of identification and their demographics (name, date of birth, phone number, email, address, race/ethnicity). The study is then described to them with an opportunity to ask questions, and informed consent is obtained. Participants can return for repeat COVID-19 testing up to a total of 55 times per calendar year with no appointments needed. Eligibility criteria include speaking English or Spanish, providing informed consent or having a surrogate provide consent. There are no age restrictions; for children 7 years and older, child assent is obtained. This study is conducted under a protocol approved by the University of California San Diego Institutional Review Board.

### COVID-19 testing workflow

**Fig. 1:**
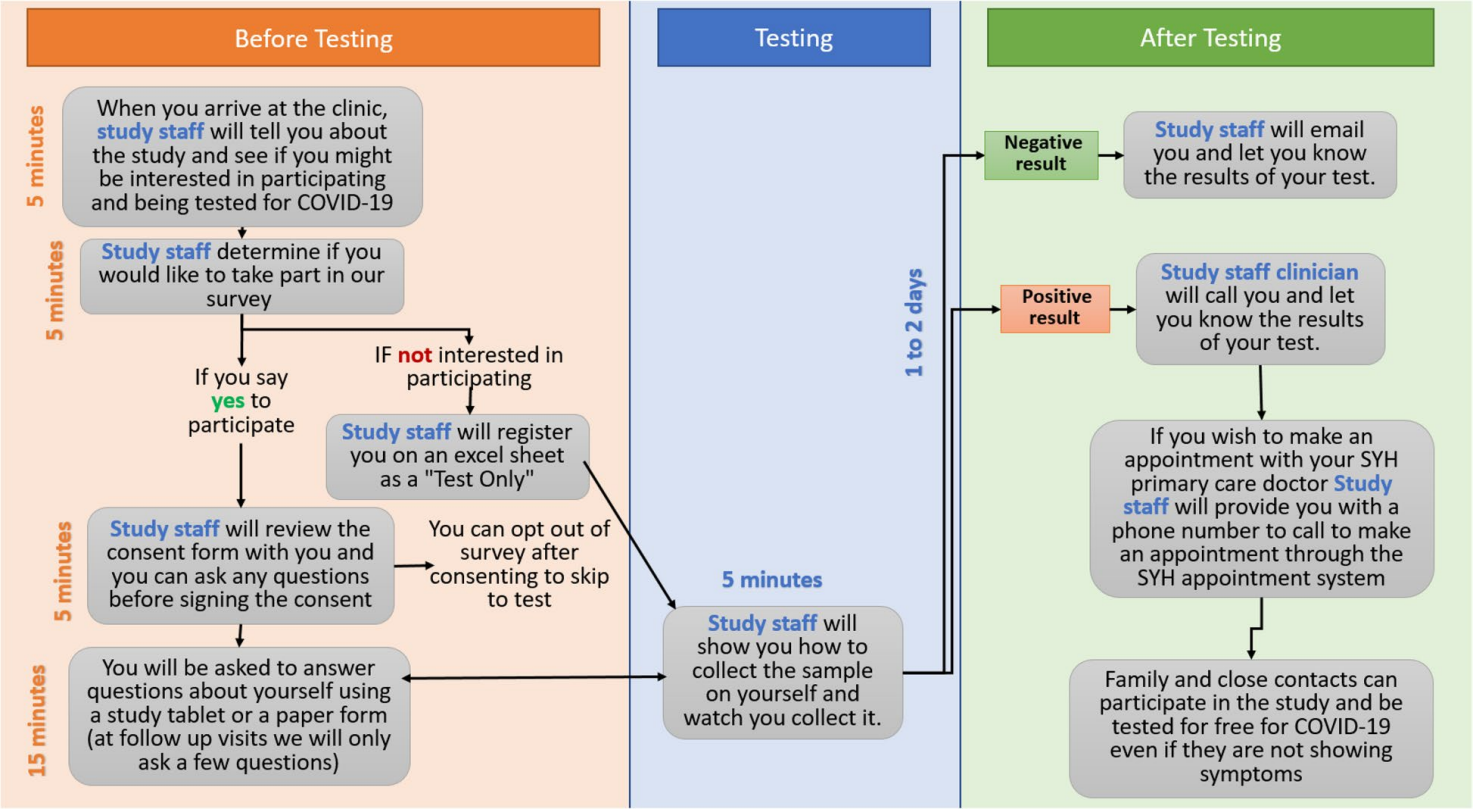
Workflow for COVID-19 testing program for patients

**Fig. 2:**
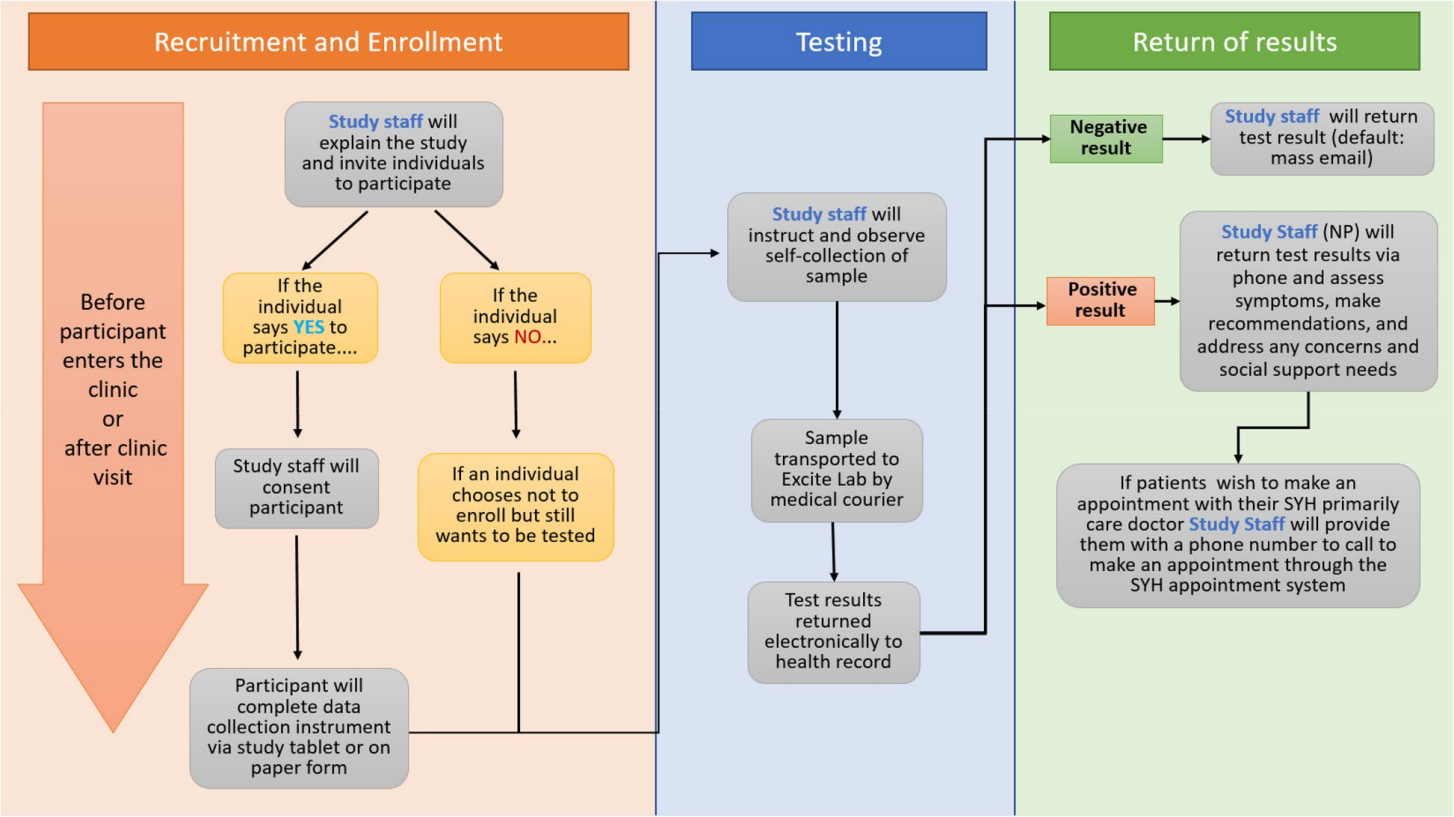
Workflow for COVID-19 testing program for providers

The initial workflow for COVID-19 testing was co-created by the research and clinical partners prior to the launch of the CO-CREATE testing program. The workflow was presented from two different perspectives to the two types of participants in the brainwriting process – one for patients (Fig. 1) and one for providers (Fig. 2). The two versions of the workflow contained information about the activities involved in the testing process which included sample self-collection, informed consent, completion of study surveys and return of results. However, they differed in how they presented the details of these activities, focusing on the patient or provider perspective and emphasizing the aspects of the program the specific participant would experience. A written narrative and narrated video describing each version of the workflow were also created in English (for providers) and in English and Spanish (for patients). The workflow was updated by the research team when major modifications were made based on feedback from research staff or patients and providers during interviews.

### Brainwriting premortem interviews (present study)

Patients and providers were interviewed in two phases: Phase 1- the early implementation phase from February 2021 – June 2021 and Phase 2—the mid-implementation phase from October 2021 – December 2021. Patients and providers were recruited using a variety of methods, including presentations at clinic staff meetings, patient contact lists, and on-site recruitment at the COVID-19 testing site. The brainwriting premortem interview guide (Table 1) was developed based on methodology by Gilmartin et al [23]. and refined for this study with three main sections: 1) general background about the participant; 2) brainwriting premortem exercise to identify key reasons why the COVID-19 testing program as described would fail; and 3) solutions for the identified COVID-19 testing program failures. To be eligible as a patient participant, individuals were required to be adults (18 years or older), speak Spanish or English, and be either a clinic patient or someone who was a close contact, family member of a clinic patient. To be eligible as a provider participant, individuals needed to be adults (18 years or older) and employed as a clinical provider or administrator at any of the clinic’s primary care facilities. Participants were provided a $40 gift card for completing an interview. Interviews were conducted in-person or virtually using Zoom in Spanish or English, lasted 15–30 min, and were recorded. CO-CREATE study staff conducted the interviews after participating in a training led by experts in qualitative data collection methods. The training included brainwriting premortem literature review, interactive interview practice sessions, and developing detailed protocols. All interviewers observed the first interview (with a provider) as part of their training that was conducted by an experienced clinical research interviewer. The team met after to discuss and refine the process.

**Table 1:** Brainwriting Premortem Interview Guide

	Patients	Providers
**General background**	**“***My first set of questions will ask about general experiences accessing or receiving care at the clinic*.” 1. How long have you been receiving care at the clinic? 2. From which types of providers have you received care here? 3. On average, how many times per month do you visit the clinic to receive care for you or a close family member or friend? 4. How has this number changed during the COVID-19 pandemic [since March 2020]?	*“My first set of questions will ask about general experiences in providing care at the clinic*.” 1. How long have you been working at the clinic? 2. [If a provider] On average, how many patients do you see for ***in-person*** visits each week? 3. How has this number changed during the COVID-19 pandemic?
**Presentation of COVID-19 testing program workflow**	Fig. 1	Fig. 2
**Brainwriting premortem**	Now, please take 5 min to think about this proposed program and why you think it might not work for you or other community members who receive care at the clinic. If you can, write down any thoughts so we can discuss them and let me know when you are ready. *(Keep flow diagram on screen for the participant to reference).* Let’s start by reading through the list. Now I’d like you to identify which are the **top three** most important reasons from this list? 1. Let’s start with what you think is the most important reason for failure? 2. Do you have suggestions or ideas about how to address this failure? 3. Let’s move on to another reason for failure. What is that? 4. Do you have suggestions or ideas about how to avoid or address this failure? *Repeat for up to three reasons or until 30 min have elapsed*	Now, please take 5 min to think about this program and why you think it might not work for you, your patients, and/or other community members who receive care at the clinic. Think about what the key challenges and barriers may be for implementing this program at the clinic as well as the population that it serves. If you can, write down any thoughts so we can discuss them and let me know when you are ready. *(Keep flow diagram on screen for the participant to reference).* Let’s start by reading through the list. Now I’d like you to identify which are the **top three** most important reasons from this list? 1. Let’s start with what you think is the most important reason for failure? 2. Do you have suggestions or ideas about how to avoid or address this? 3. Let’s move on to another reason for failure. What is that? 4. Do you have suggestions or ideas about how to avoid or address this? *Repeat for additional ideas*

After consenting, participants provided general background information and were then introduced to the appropriate version of the COVID-19 testing workflow (patient or provider; Figs. 1 and 2). The testing flow was presented visually while a pre-recorded video of the interviewer was played to verbally describe the process. Participants were asked to imagine that the testing program failed and to think about why it failed. They were asked to list all potential reasons for the program’s failure and then to identify what they thought were the top three reasons for failure. Interviewees were then asked to identify possible solutions to their reasons for failure, as well as anything else they wanted to share or general comments. Interviewers took notes during the interviews about actionable solutions that could be implemented immediately (e.g., posting location and time of testing offerings on social media sites). The COVID-19 testing program workflow was modified for Phase 2 based on solutions that were presented during Phase 1 interviews as well as research staff recommended changes from early implementation. Phase 2 interviews (October 2021 – December 2021) showed participants this updated workflow but the interview questions remained the same.

### Analysis

After interviews were completed, the recordings were professionally translated and transcribed. Data cleaning was completed by research staff. A rapid thematic analysis approach was used to identify overarching themes from the interview transcripts. Rapid thematic analysis is an applied but still rigorous method that can be used to produce actionable and targeted information on a shorter and more pragmatic timeline than traditional thematic analysis. This approach can be used in circumstances when there are highly structured and defined deliverables and the information gained from the analysis is meant to inform specific actions (i.e., refining a program to address concerns raised by diverse partners) [29–31].

Coders were trained by an expert in this data analysis approach. Interviews were initially double-coded using pre-determined codes based on the interview guide and reviewed together to build consensus and resolve discrepancies between coders. A second round of coding was conducted by the lead coder (BR) who synthesized all codes and identified additional subthemes and topics which were then verified by secondary coders (LA and KC). Data were organized by respondent type (patient and provider) and time point (Phase 1: early implementation and Phase 2: mid implementation) to explore differences and similarities across individuals and time points.

## Results

### Characteristics of patient and provider participants

A total of 11 patient interviews (5 in the early-implementation phase; all conducted in Spanish) and 6 in the mid-implementation phase (2 conducted in Spanish, 4 in English) and 8 provider interviews (4 in each phase; all were conducted in English) were completed. Participants between the two phases did not overlap. Patients reported being clinic patients for a few months to 20 years, and 3 reported not being a clinic patient and visiting the clinic for the purpose of testing. Patients reported seeing a variety of providers and number of clinic visits ranged from rarely to as many as 10–11 per month. Approximately half of the patients reported a decrease in visits since the COVID-19 pandemic started, half reported an increase in visits, and one person reported no change. It was also noted that the types of visits changed with the COVID-19 pandemic (e.g., more telehealth visits compared to in person visits).

Providers reported their tenure as ranging from 1 to 35 years in Phase 1 (*mean* = 14 years) and 1 to 16 years in Phase 2 (*mean* = 6 years). Fifty percent of Phase 1 providers were in the Pediatrics department, while 25% were in OBGYN, and 25% were in another department. Seventy-five percent of providers in Phase 2 were in Nursing and 25% in Adult Medicine. Most providers in Phase 1 were Physicians (75%) and 25% were in Clinical Administration, while in Phase 2, most were Clinical Administration (75%) and 25% were Clinical Staff. Providers reported between 40–100 patient visits per week pre-pandemic with most providers noting a decrease in in-person patient visits since the start of the COVID-19 pandemic due to an increase in telehealth visits. It was also noted that adult in-person visits were more impacted compared to pediatric visits and that in-person visits with RNs were higher compared to provider visits.

### Top reasons for COVID-19 testing program failure

Some of the themes and subthemes span across both implementation phases and participant groups. The 6 main themes that emerged from the reasons for the potential failures of the COVID-19 testing program in both early and mid-implementation were advertising/sharing information, handling of test results, staff and patient safety and testing options offered. In contrast, patient beliefs and views of the SARS-CoV-2 virus emerged in early implementation only and access to testing emerged in mid-implementation. (Table 2). Access to testing was the most commonly mentioned failure by patients and providers and emerged in Phase 2.

**Table 2:** Themes for Top Reasons for COVID-19 Testing Program Failure from Patient and Provider Interviews during Early Implementation and Mid-Implementation Phases

Patients		Providers	
**Phase 1/Early Implementation** Themes *(n)* - Subthemes	**Phase 2/Mid-Implementation** Themes* (n)* - Subthemes	**Phase 1/Early Implementation** Themes *(n)* - Subthemes	**Phase 2/Mid-Implementation** Themes *(n)* - Subthemes
**Advertising/Sharing information** (*n* = 3) - Not doing enough publicity/promotion - Brochures/information sheets having too many words, too much to read *"I think, not doing enough publicity, that wouldn’t help them either. I feel that people need to be informed about the tables or tents being there, that everyone is informed, because many people don’t know about it.”*	**Access to testing** (*n* = 4) - Hours are limited (*n* = 2) - Not being available when need to get a test - Not having enough or weekend days *"the number one is the hour of operation. I believe during the week it's from 8:00 to 3:00. Yeah. So I feel those hours are difficult for people to come out and get tested because of their work."*	**Handling test results/Return of results** (*n* = 4) - Hard time reaching patients (*n* = 3) - Extra work for staff if need to deliver results at in-person appointments *"the third and final thought was just imagining that follow-up for positive results may be unreliable. Phone numbers are not always as reliable for our population. And what do we do with a positive result and an inability to get a hold of [them?]"*	**Access to testing** (*n* = 7) - Testing locations not convenient or safe from cars driving by (*n* = 3) - Not having extended or weekend hours (*n* = 2) - Interfering with patient access to care due to parking and logistics - Lack of technology literacy/access *“I think just access to testing. And what I mean, access to testing, it's just the time that you're available to test and not being limited to just daytime hours, but opening it up to extended hours or PM hours in addition to weekend access as well….this is a working-class community. A lot of people are commuting to work or in work or commuting back home during those times. So it could be a little difficult for somebody that needs to seek care after work or before work, and not having that available could be a deterrent.”*
**Safety** **–** Patients (*n* = 2) – not following COVID-19 safety protocols – testing location near the main clinic entrance might make others fearful of being exposed *"even seeing the cases of the deceased or deaths, they don’t follow the care protocols. So since people don't believe, and don't follow the protocols of care, I think that can lead to the failure of the samples or tests that you might do."*	**Handling test results/ Return of results** (*n* = 3) - Not knowing how to complete form if don’t have US address - Not knowing how to complete form if don’t have email - Mistrust of emailed test results due to formatting *"I notice that some of the formatting is different on the emails that you guys send out to give the results. Right? I mean pretty much, it's the same. But when you guys either format the date of birth or put the name, it's a little bit different. So I feel if you guys had a more– if only you guys agreed on one basic template and used that template that just throw in everybody's name and date of birth, that'd be easier, because I can see how elaborate some of the these confirming those results would be like, "Why are those results different?" They're all the same, but why is their name or date of birth formatted in different areas so it looks like they're manipulated? When in fact they're not."*	**Patient beliefs and views regarding the SARS-CoV-2 virus** *"And others that have already been infected by COVID and believe that they have natural immunity; thus, no need to be vaccinated or tested."*	**Safety** (*n* = 3) - Patients (*n* = 2) - being around potentially infected people - not maintaining social distancing during testing process - Staff (*n* = 1) - not enough protective equipment *"see if there's patients who are symptomatic versus patients that are not symptomatic or people that are doing this for travel, there's less risk of them having it. So that could be a potential like, yes, they can do the survey, but for the other ones, I would say maybe after they've received a negative result, we could re-initiate the survey just to make sure because I think at one point we noticed there is a lot of people just in the area where the survey was…And there's also another red flag regarding too many people and the fact that they just got tested that they're not sure if they have COVID or not."*
**Patient beliefs and views regarding the SARS-CoV-2 virus** (*n* = 2) *"I think, more importantly, it’s the people who are apathetic, who don’t believe in this virus, who don’t trust it, who believe that there are, for example, political reasons to distract people. I think that’s the most important thing, there are many people who do not believe it, who believe that it’s something temporary"*	**Advertising/Sharing information** - Not doing enough publicity *"one of them is I didn't know about the program until here, so I don't know if this is something that is being promoted somewhere else. "*	Difficult consent process *"So the first thing I noticed is that the consent process could be difficult, right in the beginning, getting consent to enter the study."*	**Handling test results/Return of results** (*n* = 2) - Return of results not timely - Not communicating positive results to provider *"Turnaround time on test collection data. If it's taking more than 24 h to get a test result back, it could cause somebody not want to refer or to return if symptomatic, for a particular reason, just because they need results in a sooner manner"*
Free testing sends message that it is not good quality *"And, with having free testing, like Latinos always believe that something is bad, like it won’t be good quality, when it’s something for free. That would be the reason, that they don’t trust, that’s contradictory, because first it’s too expensive, and you don’t have the money to pay for it, but then they offer it to you for free and you distrust it. In Mexico we say, “I don't want to be the guinea pig,” that’s, I don't want to be the test animal, for example. When it's free, people think of it that way."*	Clinic location in parking lot might seem untrustworthy (for those with Latino backgrounds) *"I think having a testing site out in the parking lot can seem a little bit untrustworthy. I mean, yeah, it's next to a clinic. It's next to a licensed building with just doctors and everything, but having it out in the parking lot can seem a little untrustworthy for other people coming from a Latino background"*	Cost of appointment for patient to discuss positive results *"And essentially, it's going to be difficult for a newly registered patient who has no funding to complete that part of the system. Because when we do a telephone visit, our receptionists call up and say, we're doing this telephone visit, there's a fee. Do you have medical insurance? If you don't, then there's a fee. So at that point, even though every step before that would be free, the clinic's not going to do telephone visits for free, so."*	**Advertising/Sharing information** (*n* = 2) - Not doing enough publicity *“If we're not promoting enough awareness to the testing access or to the testing activities, that could be a barrier as to why it would not be successful….promotion of the services, what's being done in the community to really get the word out that this is available.”*
Misinformation from unreliable sources *"..think this is one of the main problems, that really, the news, the way the information reaches us or the information is downloaded [inaudible], many times it’s not that reliable. Unfortunately, social media right now– people let themselves be guided too much by social media, instead of taking into consideration if it’s true, or that it’s a way of spreading information, rather than being something true on social media, on Instagram, not from Twitter, not from Facebook. "*	Staff member needed to supervise on site logistics and supplies *"that there be a person supervising that, who’s on top of that, “Let’s see, what’s lacking? Know what? We’re lacking–,” whether that be tarps or more medical equipment, more chairs, more security. That somebody’s on top of that.*"	Culture, fears, beliefs of patients *“Culture is a big one. Here being predominantly a Mexican community, the culture here is, in a sense, very traditional. And I want to say that there's skepticism within the culture. And it is a barrier that I think the health care in general has to work to continue getting people over the barrier of them saying, "Well, in my culture, we wouldn't necessarily take our kids to get tested for this or that. We're going to stay home and do home remedies."*	Patients feeling ill and not wanting to participate *"if someone's ill or under the weather, that may be a factor, too, in terms of them wanting to participate. They're there for care or they want to get that care and then be on their way"*
Patients might think testing is not free as early tests were expensive *"despite the fact that here, you’re trying to make them free, the tests, for example, the first tests were very expensive, access to the COVID test was too expensive, so people didn’t do it. But I believe that we have, unfortunately, no way to make them aware that it does exist, and that it’s necessary to carry out these tests."*		Interfering with patient access to care and satisfaction due to increased documentation/paperwork *"the intake and everything could potentially interfere with general patient flow for their visit, delaying them getting registered for their visit. There can be quite a lot of paperwork that they have to do just around a physical. Often their insurance may not be active, it has to be– so it's already a slow process from the time they hit the door to the time they see the physician. And if we're throwing more questionnaires and obstacles in that way, it may delay their experience at the clinic to a point where it– past the tipping point of them being satisfied with their experience…"*	Survey length too long and reading level too high *"in terms of survey, just the length of the survey, 50 questions seems like a lot to have to go through because you do have to spend some time to really read and understand the questions. And I don't know at what literacy level the questions are being promoted at"*
Process not being agile and efficient *"there are a lot of people who are very impatient, so it could be, like, if the process were agile, so that it could get done. That’s the most important, because I’ve had to go to certain places, and there are many procedures, and it’s very time consuming, so make it agile, I think that’s the important thing for the patients."*		Lack of trust in system and science by patients *"Also a lack of trust in the system by some. … Well, not only from what I see, of course, on the news, but the patients that we do speak to, those who are being hesitant, there is a lot of conversation about how trustworthy science is and, specifically, this vaccine. Everyone is talking about how it was developed so quickly, all of the different conspiracies. And so patients come in with their stories. And they field a certain believe that creates a barrier, and it does not allow them to trust, in this case, the health care ystem. So they brushed it off. And that's why I feel that there is a level of lack of trust in the system, the government. In the science."*	**Testing options offered** - not having options such as rapid antigen tests *"the testing options that are available at the test site, so maybe rapid antigen testing, and give options in terms of what the patient may want– or what the patient has available."*
Religious beliefs of patients *"Yes. Religion, religious beliefs, I think of that. I mean, there are an infinity of cults that don’t allow getting vaccines, even as a baby, so right now, I think that one, also."*		Ordering of tests process is not that easy *"Who is actually going to order the tests? Someone has to be on staff here to be able to order a test and have set access to order tests through our system. It wasn't clear to me whether the test was being ordered through Cocreate or was it being ordered through our system. In our system we're going to have that next gen access and figure out how to do that. It's not that easy. "*	
		Registering patients on system can be time consuming *"The idea of newly registering a patient in five minutes is very optimistic. I can tell you that happens all the time with us. That would be very optimistic. And often they don't really get assigned even their newly registered right to a primary care physician. So we'd have to make sure that that happens."*	
		**Testing options offered** - parents might be reluctant collecting sample for their children *"So the first one was just self-collection and whether parents would be comfortable doing that for their young children, especially an infant or a toddler. I'm assuming it's an anterior nares swab that you're having them do, and that's not that invasive, but they still would potentially be reluctant to do that"*	
		Vaccination leading to no need for testing *"the research may fail because of the vaccine because people are getting vaccinated. We're really pushing the vaccine and we are now pushing for 12 and over. So the study may fail because of just the vaccine"*	

**Bold** text indicates a theme repeated across time and/or patients and providers

*N* indicates the number of times mentioned

Subthemes for advertising/sharing information included not promoting the testing program enough and the brochures and study information having too much to read/too many words. Subthemes that emerged for access to testing were limited hours/days and no weekends, inconvenient testing locations, unsafe testing locations, and lack of technology literacy/access to participate. Subthemes for handling of test results were concerns regarding the completion of forms without a U.S. home address or email address, mistrust of emailed test results, difficulty reaching patients with their test results, extra work for staff if results were delivered in person, lack of timely return of results, and not communicating positive results to patients’ providers. Subthemes for safety included patients not following COVID-19 safety protocols, patients being in the presence of potentially infected people at the testing site, the fear of being exposed because the testing location would be near the main entrance to the clinic, and insufficient personal protective equipment for staff. Both patients and providers listed patient beliefs and views of the SARS-CoV-2 virus as a reason for failure, and providers also noted that limited testing options offered would be a reason for failure, specifically parents being reluctant to collect samples for their children and the lack of rapid antigen testing since, at the time, CO-CREATE only offered polymerase chain reaction (PCR) tests. Other themes that emerged as reasons for program failure during Phase 1 only included misinformation from unreliable sources, culture, fears and beliefs of patients, lack of trust in the system and science, and religious beliefs. These themes did not emerge in Phase 2 and were replaced by more pragmatic themes related to access to testing and handling of test results.

### Proposed solutions to COVID-19 testing program failures

The main themes that emerged from the proposed solutions to the COVID-19 testing program failures overlapped with the perceived failures in both early and mid-implementation included advertising/sharing information, access to testing, handling of test results, and testing options offered while education emerged in early implementation only and staff safety, physical operations, and recruitment strategies emerged in mid-implementation (Table 3).

**Table 3:** Proposed Solutions to COVID-19 Testing Program Failures from Patient and Provider Interviews during Early Implementation and Mid-Implementation Phases

Patients		Providers	
**Phase 1/Early Implementation** Themes *(n*) - Subthemes	**Phase 2/Mid-Implementation** Themes*(n)* -Subthemes	**Phase 1/Early Implementation** Themes* (n)* -Subthemes	**Phase 2/Mid-Implementation** Themes *(n)* -Subthemes
**Advertising/sharing information** - Clarity about when and where testing is offered	**Advertising/sharing information (***n* = 6) - Provide visual pieces describing the testing program at testing site (*n* = 2) - Publicize when and where testing is offered on website or social media - Use TV, flyers, schools/parents, word of mouth to publicize - Make information very specific and short - Explain the study to people in writing	**Education** (*n* = 2) - Provide a hands-on demonstration of the testing process to show it is harmless - Provide education about testing and vaccination	**Safety (Staff)** (*n* = 4) - Provide N95s for those in front line contact with symptomatic patients - Designate a place for PPE donning and doffing - Set up separate testing areas for symptomatic and asymptomatic test-seekers - Collect surveys from symptomatic patients after their quarantine period
**Education** - Help people understand the severity of the COVID-19 pandemic	**Access to testing** (*n* = 5) - Offer extended hours (*n* = 3) - Offer Sundays, with no appointment - Move testing location to a more secure, safe, private space indoors	**Access to testing** - Locate testing site in front of clinic	Physical operations (*n* = 3) - Use floor stickers to designate 6 feet distance for those standing and moving in line - Use signs to indicate where to go and/or barriers so people can't go another way - Stock up on supplies when a surge is expected
**Handling test results/ Return of results** - Send test results by email	**Handling test results/ Return of results** (*n* = 3) - Send test results by text - Communicate with the patient’s doctor - Suggest to patients they should set up an email account if don’t have one	**Handling test results/ Return of results** - Emphasize the importance of being able to get hold off people to give test results	Recruitment strategy/communication with patients (*n* = 3) - Disclose what the survey is, what it will be used for, and how long it will take up front to build trust - Share how participation will help someone else - Speak to people on their level, not coming down from a position of authority, and use words they understand
Provide testimonials from people who had COVID-19 infection to convince others	Introduce the survey with a typed half sheet of paper so patients are prepared when they go to the check in table with questions	Order tests through study staff, not through the health center system	**Advertising/sharing information** (*n* = 2)
	Randomize the survey item order	Provide free follow-up appointments/calls to patients with positive results for those with no insurance or newly registered	**Access to testing** – Offer extended hours and weekends
		**Testing options offered** – Provide saliva tests for pediatric patients	**Handling test results/ Return of results** - Send positive results to patient’s provider in an email
			**Testing options offered** - Provide rapid antigen tests

**Bold** text indicates a theme repeated across time and/or patients and providers

N indicates the number of times mentioned

Specific strategies for the theme of advertising/sharing information included incorporating visual materials describing the program available at the testing site, website/social media showing the testing schedule, using multiple modes of advertising (TV, flyers, schools/parents), and clarity about when and where testing was offered. Solutions related to advertising/sharing information were the most mentioned and emerged in patients for both Phases and providers only in Phase 2.

Specific strategies for access were extended hours and weekend testing, walk-up testing, easy access to testing at the clinic, moving testing to a more private location indoors, and having the testing site in front of the clinic. Recommended strategies for handling test results were: sending results by email to patients and their providers to communicate positive results, emphasizing during testing the importance of accurate contact information so that participants could be reached with test results, sending results by text, and encouraging patients to set up an email account if they did not have one. Solutions related to access to testing and handling of test results were commonly mentioned and emerged in both phases for patients and providers. Strategies for testing options offered were: providing a saliva test for pediatric patients and having rapid antigen tests available. Solutions related to testing options offered emerged with providers only, in both phases. Strategies for education highlighted helping people understand the severity of the COVID-19 pandemic, education about testing and vaccination, and a hands-on demonstration of the testing process. Specific strategies for staff safety were: N95s for those in front-line contact with symptomatic patients; designated places for personal protective equipment donning and doffing; separate testing areas for symptomatic and asymptomatic test-seekers; and delaying research surveys for symptomatic patients until after their quarantine period ended. Strategies for physical operations were: using floor decals to designate 6 feet distance for lines; posting signs to indicate flow; and stocking up on testing supplies when a surge is expected. Finally, strategies for recruitment were: disclosing the content and purpose of the research survey at the start of the testing process; sharing how participation will help others; and speaking to people in respectful and appropriate ways based on their level of literacy and comfort. The safety, physical operations, and recruitment themes were only mentioned by providers in Phase 2 interviews.

### Changes to program flow based on proposed solutions

A number of changes to the testing workflow were made in response to solutions proposed by patients and providers during the brainwriting premortem interviews (Table 4). Changes related to the handling of test results were the most common and included sending results via text with distinct workflows based on the test result. Specifically, all results were sent to patients by text, but a request to contact the team was sent only to those with positive results (patients with negative or invalid results were not asked to contact the team). Patients with positive results were also called by a physician or nurse on the research team, with three attempts made.

**Table 4:** Proposed Solutions to COVID-19 Testing Program Failures and Changes Made to Program Flow to Address Them

Proposed Solution for COVID-19 Testing Program Failure	Implementation of Proposed Solution
**Patients**	
**Advertising/sharing information –** Provide visual pieces describing the testing program available at the testing site	Aesthetically pleasing flyers are provided at the testing site, website, and social media.
**Advertising/sharing information—**Publicize when and where testing is offered on website or social media	On-site staff update the website and social media as needed in real-time instead of emailing off-site volunteers to help.
**Advertising/sharing information –** Use TV, flyers, schools/parents, word of mouth to publicize	Interns distributed flyers throughout the community in the winter of 2021. The testing program was featured on news TV and outreach was conducted at all local schools and private schools informing them of testing services.
**Handling test results/ Return of results**—Send results by text	Results are sent via text if they are negative or invalid and those with positive results are texted with a message to contact the CO-CREATE team to get results by phone.
**Handling test results/ Return of results –** Communicate results with the patient’s doctor	Patient test results are sent to clinic medical records to be imported into patients medical charts. Patients with positive test results are encouraged to contact their Primary Care Physician for follow-up.
**Handling test results/ Return of results**—Send test results by email	Results are being sent via email as the priority.
**Education**—Help people understand the severity of the COVID-19 pandemic	CDC/CDPH COVID-19-related flyers and information has been made available.
**Providers**	
**Handling test results/ Return of results**—Emphasize the importance of being able to get a hold of people to give test results	We ask for multiple methods of contact information (phone, email, address).
**Handling test results/ Return of results**—Send positive results to patient’s provider in an email	We send results to the clinic via secure document.
**Safety (Staff)** – Provide N95s for those in frontline contact with symptomatic patients	All staff are required to use KN95's on-site instead of normal face masks.
**Safety (Staff)**—Collect surveys from symptomatic patients after their quarantine period	We encourage all participants to take the survey home and only return the survey once they receive their negative results or if they receive positive results, when they complete their isolation period.
**Education** – Provide education about testing and vaccination	Educational forms are available on-site, on our website and social media for patients to review.
**Education** – Provide a hands-on demonstration of testing process to show it is harmless	We ordered a model nose to show how far the swab goes inside the nose and how easy it is for people to collect their own sample.
**Physical operations –** Use floor stickers to designate 6 feet distance for those standing and moving in line	We started this during the 2022 Omicron surge, using colored cones to help distancing.
**Physical operations –** Use signs to indicate where to go and/or barriers so people can't go another way	We tried this, our community doesn't follow.
**Recruitment strategy/communication with patients**—Disclose what the survey is, what it will be used for, and how long it will take up front to build trust	This has been implemented since the beginning of the project.
**Testing options offered** – Provide rapid antigen tests	We started offering antigen testing for the community in June 2022.
Order tests through study staff and not through the health center system	We cut the time per participant in half by using a secure electronic database to transfer test tube barcodes rather than ordering tests through the clinic.

Changes to the advertising/sharing of information and education were the second most common, resulting in the development and distribution of aesthetically pleasing flyers at the testing sites. Designated on-site staff managed social media and website updates instead of relying on off-site volunteers to help with updates. Changes were also made to solutions proposed for staff safety (e.g., requiring N95s for on-site staff instead of normal face masks), physical operations (e.g., using colored cones to help with social distancing when patients stand in line for tests), and testing options offered (e.g., provided rapid antigen tests starting in June 2022). Overall, changes were made in response to 7 suggestions from patients and 9 suggestions from providers.

### General comments

Participants were prompted to share general feedback or comments and positive feedback was noted as a common theme by English-speaking patients. This included: viewing the testing program as easily accessible and a good resource for the community; noting that trust was built in the community by testing everyone (symptomatic and asymptomatic); agreeing with focusing on areas with high positivity rates; and noting that test results were delivered in a more reliable and efficient manner with CO-CREATE compared to other testing services. Spanish-speaking patients commented that incentives to participate in the study and the outdoor testing location were good, in addition to also viewing that the testing program as easy to access and acceptable. Providers commented in Phase 2 that the testing program was a good resource for the community, that CO-CREATE staff were helpful, courteous and communicative, that the testing site was located in a convenient location, and that being walk-up, no appointment, was helpful, although there were long wait times during COVID-19 surges.

## Discussion

We used partner-engaged interviews based on an adapted version of the brainwriting premortem technique to inform the iterative refinement of a COVID-19 testing program at a FQHC in the San Diego community. Multiple perspectives from providers and patients were represented in two distinct implementation time points allowing for an iterative and multi-partner improvement of the testing protocol. The process allowed us to incorporate the perspective of key partners in the delivery and iterative refinement of the COVID-19 testing program. Multiple changes to the testing protocol were made as a result of this feedback.

The most common potential failure reasons reported by patients and providers proved to be the sharing of results from the COVID-19 test with patients in a timely manner using an approach that was meaningful, low burden, and did not overextend an already overburdened healthcare system due to the pandemic. The most commonly recommended solution was related to providing information to the community about the continuously changing testing guidelines and the pandemic, especially in regard to reaching out to patients promptly and reaching all who might be interested in services offered by the testing site. Revisions to the workflow were adopted based on these recommendations. Our findings in terms of specific barriers and general recommendations are similar to other recent publications on the implementation of COVID-19 testing programs [32–35]. A broader theme that seems to be especially relevant across studies is the need to consider the preferences and circumstances of the specific priority population in a dynamic manner (at multiple time points to account for the rapidly changing pandemic context).

Engagement of partners in the creation of public health solutions has been broadly recommended in the field. Equally, if not more important, is ongoing engagement of these partners (checking in more than one time) especially when dealing with public health emergencies where guidance and evidence rapidly evolve. In a recent publication, Eisman and colleagues emphasized that rapid adaptations responding to urgent public health crises are critical to implementing impactful solutions [36]. Øvretveit also emphasized the need to use implementation science approaches to address COVID-19 pandemic challenges in health care settings [37].

As noted in the introduction, there are multiple techniques that can be used to achieve meaningful engagement of partners to inform potential program barriers and facilitators and eventually lead to strategies to address these barriers before and during implementation [17–21]. Our paper intended to expand the implementation science toolkit with a novel, less frequently used approach that happened to be also rapid. We believe that researchers should consider various approaches and select the one that seems to fit best for their circumstances. The use of the brainwriting premortem exercise was feasible to complete in the context of our study, but it was not used as a stand-alone strategy to engage with our partners. We used brainwriting premortem in combination with monthly community and scientific advisory board meetings to develop a theory of change of COVID-19 disparities in our setting [11] and an iterative ethnographic assessment of the engagement process in the advisory board meetings [16]. Jolles-Perez and colleagues recently operationalized five principles of a co-created collaborative process to enhance implementation efforts that encompass equity, reflexivity, reciprocity and mutuality, transformative and personalized, and facilitating relationships [38].

Important limitations for this study included the use of an individual instead of a group-based approach to the brainwriting premortem process. Although we were not able to capitalize on the group-based strength of the method, conducting individual interviews allowed for this approach to be feasible in the context of the COVID-19 pandemic where bringing together groups of providers and community members presented a substantial challenge. We also conducted only two waves of the brainwriting premortem process, which limited us to input at two time points (pre-early implementation and mid-implementation). In future studies, more frequent use of this technique in conjunction with other methods of implementation and evaluation could provide real-time guidance from key partners on how the program is implemented. There is increasing interest in the field in using real-time input from partners to guide and adjust implementation. Recent publications describe methods to guide this process [39].

## Conclusions

This study adds to the growing literature on pragmatic methods for rapid and meaningful partner engagement by presenting successful application of the brainwriting premortem approach to identifying and implementing revisions to a COVID-19 testing workflow during both pre- and post-launch of testing activities. Brainwriting premortem was an effective tool in the iterative refinement of a COVID-19 testing program in the context of an FQHC and can be a promising and approach to incorporate iterative input from patients and providers to ensure successful program implementation.

### Supplementary Information

**Additional file 1: Supplemental Table.** General Information about Patients and Providers from Interviews.

## Data Availability

The datasets generated and/or analysed during the current study are not publicly available due to the majority of the data being qualitative in nature, so there are restrictions to sharing to preserve the privacy of individuals. However, the co-author team will review data requests and that data will be made available as reasonably appropriate. Contact the corresponding author for data requests.

## Abbreviations

CO-CREATE: Community-driven Optimization of COVID-19 testing to Reach and Engage underserved Areas for Testing Equity—in Women and Children
RADx-UP: Rapid Acceleration of Diagnostics-for Underserved Populations
FQHC: Federally Qualified Health Center
PCR: Polymerase Chain Reaction
