# Supplementary material for: Adaptation of the brainwriting premortem technique to inform the co-creation of COVID-19 testing strategies in underserved communities in South San Diego
Source: BMC Health Serv Res. 2024 Jan 3;24:12. doi: 10.1186/s12913-023-10341-w (PMC10765849; doi:10.1186/s12913-023-10341-w)
Supplement: Supplementary file 1 — Additional file 1: Supplemental Table. General Information about Patients and Providers from Interviews. [file 12913_2023_10341_MOESM1_ESM.docx]

Supplemental Table. General Information about Patients and Providers from Interviews

|  | **Phase 1/**  **Pre-Implementation (n=5)** | **Phase 2/ Mid-Implementation (n=6)** |  | **Phase 1/**  **Pre-Implementation (n=4)** | **Phase 2/Mid-Implementation (n=4)** |
| --- | --- | --- | --- | --- | --- |
| **Tenure as clinic patient** | - 1 year - 3 years - several years (as caregiver) - 4-5 years - Over 5 years | - A few months to get tested - 2 years - 5 years - 7 years - 20 years - NA not clinic patient | **Tenure as clinic provider** | *M*=14 years (Range=1-35 years) | *M*=6 years (Range=1-16 years) |
|  |  |  | **Clinic department** | 25% OBGYN  50% Pediatrics  25% Other | 75% Nursing  25% Adult medicine |
| **Type of clinic provider seen** | - N/A caregiver - OBGYN - OBGYN + dental - All including dentist, maternity, pediatric, ER, non-ER - Primary + psychiatrist + psychologist + chiropractor | - N/A Different clinic - Primary + eye - Medical - OBGYN + PA - All services including prenatal, kids physicals, dental - N/A not clinic patient | **Role** | 75% Physicians  25% Clinical Administration  0% Clinical Staff | 0% Physicians  75% Clinical Administration  25% Clinical Staff |
| **Number of clinic visits** | - Once a month visits for their own care or visiting for the care of their children (n=most) - 3 times per month | - Once per year - 10-11 times per month - Depends if there is a medical problem/appointment - Family comes more often - Weekly to testing site | **Number of patient visits** | - 40 per week pre-pandemic - Close to 100 per week (n=2) | - 40 per week (in person + phone/video) - 10-15 per day - 60 per day at South Bay testing site, now 10 per day at nursing clinic |
| **Change in clinic visits during COVID-19 pandemic** | - No change to visit frequency - Decrease in visit frequency - Decrease in visit frequency with visits more spread out - Increase in visit frequency due to pregnancy, but participating only in essential care otherwise - Increase in visit frequency but indicated that these visits were conducted over the phone. | - Decrease in visit frequency (n=2) - Increase in visit frequency only due to testing - Increase in visit frequency due to employer mandated testing - Different types of appointments since the pandemic (phone and online) | **Change in patient visits during COVID-19 pandemic** | - Substantial drop in number of in-person visits (n=3) - Adult in-person visits more impacted and pediatric visits less impacted | - Increase in visits - Increase due to more telehealth visits - Decrease in-person visits due to increase in telehealth visits (n=2) - RN visits are more in-person compared to provider visits - Walk-in clinic changed to triage system to limit in-person visits - Remote work during pandemic, back in person now - Remote work during pandemic, not back because half of the clinics are shut down |
